# Emerging Pharmacological and Non-Pharmacological Therapeutics for Prevention and Treatment of Chemotherapy-Induced Peripheral Neuropathy

**DOI:** 10.3390/cancers13040766

**Published:** 2021-02-12

**Authors:** Yang Li, Maryam B. Lustberg, Shuiying Hu

**Affiliations:** 1 Division of Pharmaceutics and Pharmacology, College of Pharmacy & Comprehensive Cancer Center, The Ohio State University, Columbus, OH 43210, USA; li.10991@osu.edu; 2 Comprehensive Cancer Center, Department of Medical Oncology, The Ohio State University, Columbus, OH 43210, USA; Maryam.Lustberg@osumc.edu

**Keywords:** chemotherapy-induced peripheral neuropathy, cancer, neurotoxicity, treatment, prevention

## Abstract

**Simple Summary:**

Chemotherapy-induced peripheral neuropathy (CIPN) is a common and persistent complication of commonly used chemotherapy drugs. This article provides an overview of emerging therapeutics for the prevention and treatment of CIPN and focuses on pharmacological strategies that are derived from novel mechanistic insights and have the potential to be translated into clinically beneficial approaches. It is our contention to call for fostering collaboration between basic and clinical researchers to improve the development of effective strategies.

**Abstract:**

Chemotherapy-induced peripheral neuropathy (CIPN) is a common adverse event of several first-line chemotherapeutic agents, including platinum compounds, taxanes, vinca alkaloids, thalidomide, and bortezomib, which negatively affects the quality of life and clinical outcome. Given the dearth of effective established agents for preventing or treating CIPN, and the increasing number of cancer survivors, there is an urgent need for the identification and development of new, effective intervention strategies that can prevent or mitigate this debilitating side effect. Prior failures in the development of effective interventions have been due, at least in part, to a lack of mechanistic understanding of CIPN and problems in translating this mechanistic understanding into testable hypotheses in rationally-designed clinical trials. Recent progress has been made, however, in the pathogenesis of CIPN and has provided new targets and pathways for the development of emerging therapeutics that can be explored clinically to improve the management of this debilitating toxicity. This review focuses on the emerging therapeutics for the prevention and treatment of CIPN, including pharmacological and non-pharmacological strategies, and calls for fostering collaboration between basic and clinical researchers to improve the development of effective strategies.

## 1. Introduction

Chemotherapy-induced peripheral neuropathy (CIPN) is among the most common adverse effects of chemotherapy, affects several millions of patients each year, and is characterized by tingling, numbness, increased sensitivity to cold and touch, and burning pain of the distal extremities [1,2]. Although the prevalence of CIPN varies depending on the anticancer agent, dose, duration of exposure, and method of assessment, it can be as high as 89% for acute symptoms and 85% for chronic CIPN [3]. The anticancer agents most commonly associated with CIPN include paclitaxel and oxaliplatin, although the side effects have also been reported after treatment with other taxanes (docetaxel) and platinum agents (cisplatin and carboplatin), vinca alkaloids (particularly vincristine), and bortezomib. CIPN is a tremendous health problem worldwide and remains one of the most important complications of contemporary oncology regimens as it may limit further use of curative-intent treatment and/or may cause the long-term quality of life concerns [4,5].

Despite having been the focus of intense investigation for several decades, management of CIPN continues to be challenging. According to the most recent 2020 American Society of Clinical Oncology (ASCO) CIPN guideline, no agents can be recommended for the prevention of CIPN due to lack of high-quality evidence in general, which remains unchanged since the initial 2014 guideline. Although the earlier guideline commented on venlafaxine as a preventative agent, the updated guideline does not recommend it based on a negative follow-up study, in which patients were treated for a longer time-period and evaluated with a more widely accepted CIPN measurement tool [6]. The ASCO guideline also supports duloxetine as the sole treatment option for established painful CIPN, for which additional supporting data has become available since the initial guideline in 2014, but no longer supports the utility of tricyclic antidepressants, gabapentinoids, or topical amitriptyline/ketamine/baclofen that were included as therapeutic approaches in the initial guideline.

**Figure 1:**
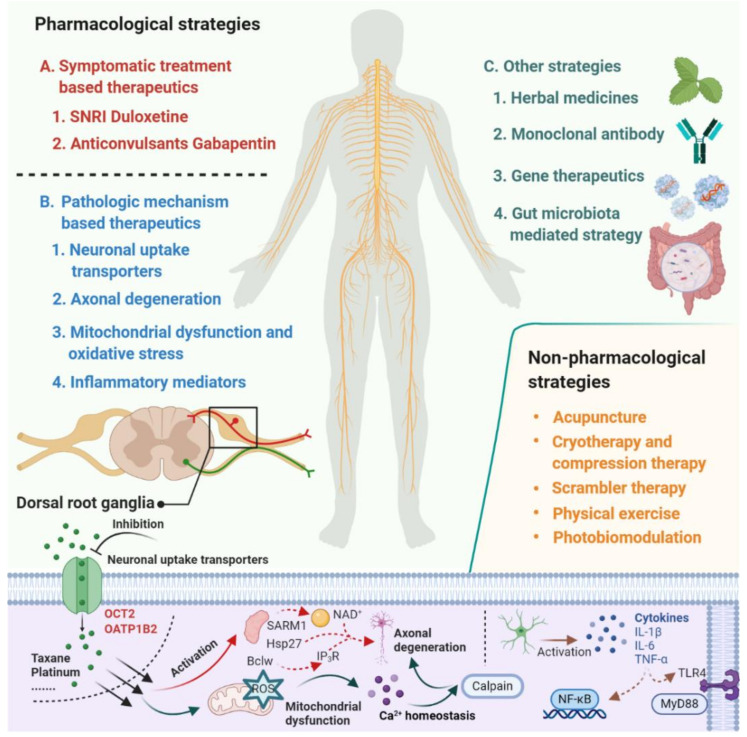
Summary of emerging pharmacological and non-pharmacological therapeutics for prevention and treatment of chemotherapy-induced peripheral neuropathy.

Despite the extensive research effort focused on understanding mechanisms involved in the development of CIPN, the translation of this mechanistic understanding into rationally-designed, clinical intervention studies remains problematic [7]. According to a study conducted by investigators at the National Cancer Institute, based on databases covering the period January 2011 to May 2019, only 3 of 35 clinical trials that investigated agents or devices to treat CIPN incorporated a mechanistic rationale to support the choice of the intervention. This not only speaks to the urgent need for collaborations between basic and clinical research teams but also emphasizes the thesis that a deeper understanding of the underlying mechanisms that initiate and cause the progression of CIPN will ultimately facilitate the development of effective prevention and treatment strategies [8,9,10]. In the current article, we provide an overview of emerging therapeutics for the prevention and treatment of CIPN and focus on pharmacological strategies that are derived from novel mechanistic insights and have the potential to be translated into clinically beneficial approaches (Figure 1).

## 2. Pharmacological Strategies for Prevention/Treatment of CIPN

There are two overarching approaches in CIPN management: to target the underlying pathologic mechanism responsible for CIPN or to address the CIPN symptoms themselves [4].

### 2.1. Pathologic Mechanism-Based Therapeutics

The pathologic mechanism by which chemotherapeutics damage structures in the nervous system and cause CIPN is multifactorial [10]. Recent progress in preclinical studies has identified several new preventative and therapeutic targets and pathways, which have the potential to be translated into the clinic for improved management of CIPN.

#### 2.1.1. Neuronal Uptake Transporters

Prior investigations have demonstrated that one of the initiating events leading to the development of CIPN is the extensive accumulation and retention of chemotherapeutic drugs in the peripheral sensory nerves present in dorsal root ganglia (DRG) [11,12]. For many small-molecule anticancer drugs, this process is mediated by uptake transporters located in DRG neurons, and ongoing efforts attempt to block these transporters, reduce intra-neuronal concentrations of the neurotoxic agents, and ultimately protect against a dose-limiting injury. The role of drug transporters in CIPN was recently extensively reviewed [13], and this field of research will be illustrated here for oxaliplatin and paclitaxel, two agents for which defined transporters have been identified that can be targeted pharmacologically.

Preceding investigations have found that facilitated transport mechanisms are responsible for the neuronal uptake of platinum-based chemotherapeutic drugs associated with CIPN such as oxaliplatin [14]. Although recent have suggested emerging importance of multiple solute carriers in oxaliplatin neurotoxicity, including the organic cation transporters novel 1 (OCTN1) and multidrug and toxin extrusion 1 (MATE1), the actual in vivo contribution of these proteins to CIPN has remained inconclusive [13]. Using transporter-deficient mouse models, it was recently suggested that the direct contribution of OCTN1 and MATE1 to oxaliplatin neurotoxicity is negligible and that the neuronal uptake of oxaliplatin and the subsequent neurotoxicity is predominantly mediated by the related transporter organic cation transporter 2 (OCT2) [15]. This transporter is highly expressed in satellite glial cells with the DRG neurons, and targeting OCT2 using genetic and pharmacological approaches can ameliorate both acute and chronic forms of oxaliplatin in mice and rats [15].

An analogous uptake mechanism in peripheral neurons has been reported for paclitaxel, and this was found to be mediated by the organic anion transporting polypeptide B2 (OATP1B2) as supported by the observation that OATP1B2 deficiency in mice was associated with complete protection against paclitaxel-induced peripheral neurotoxicity [16]. Interestingly, the function of the OCT2 and OATP1B2 transporters can be effectively blocked by several FDA-approved tyrosine kinase inhibitors (TKIs) through inhibition of kinases that mediate phosphorylation-mediated activation [17]. Indeed, functional validation studies have now demonstrated that TKIs such as dasatinib (an OCT2 inhibitor) and nilotinib (an OATP1B2 inhibitor) could be exploited as effective neuroprotective agents in treatment regimens involving oxaliplatin and paclitaxel, without affecting plasma levels of the chemotherapy or negatively influencing antitumor efficacy [15,16]. These recent studies have not only shed light on the etiology of CIPN associated with small-molecule cancer drugs but provided a direct rationale for currently ongoing clinical studies aimed at the identification of new targeted interventions using TKIs as transport inhibitors to mitigate a potentially debilitating side effect in patients receiving oxaliplatin- or paclitaxel-based treatment regimens [18,19].

#### 2.1.2. Axonal Degeneration

Direct damage of peripheral nerves by axonal degeneration is the most significant final result from CIPN. Neurofilament light chain (NfL) has been developed as a specific serum biomarker of axonal degeneration to follow the onset and severity of axonal degeneration resulting from CIPN [20]. Several recent publications have highlighted several pathways involved in axonal degeneration. In particular, it has been reported that the sterile alpha and TIR motif-containing protein 1 (SARM1) triggers axonal degeneration by inducing the rapid destruction of the essential metabolic co-factor nicotinamide adenine dinucleotide (NAD^+^), which induces metabolic collapse and axon death [21,22]. Strategies targeting this pathway are of high interest, and several preclinical studies have been performed in connection with CIPN prevention [23,24].

In addition to the SARM pathway, the small heat shock protein Hsp27 is highly upregulated after peripheral nerve injury, and upregulation of Hsp27 markedly enhances the intrinsic regenerative capacity of adult sensory neurons and accelerates axon regeneration and functional recovery. Forced expression of Hsp27 completely reverses vincristine-induced CIPN symptoms by targeting multiple aspects of CIPN pathology, including axonal and mitochondrial integrity as well as calcium homeostasis [25]. It may be worthwhile to identify small molecules that can activate key signaling pathways of Hsp27 in future studies to identify novel preventative strategies of relevance to vincristine neurotoxicity [25]. In addition to studies focusing on vincristine, paclitaxel impairs axonal trafficking of RNA-granules and reduces the synthesis of Bcl2 peptide family member (Bclw), a Bcl2 family member that binds inositol 1,4,5-trisphosphate receptor 1 (IP_3_R1) and restrains axon degeneration. The Bclw-IP_3_R1-dependent cascade causes axon degeneration, suggesting that Bclw-mimetics could potentially offer neuroprotection against agents such as paclitaxel [26].

#### 2.1.3. Mitochondrial Dysfunction and Oxidative Stress

Mitochondrial dysfunction and oxidative stress have been highlighted as key players in the pathophysiology of CIPN. Impairment of the mitochondrial physiological function is leading to increased production of reactive oxygen species (ROS) and oxidative stress [27]. Mangafodipir, a contrast agent used in magnetic resonance imaging (MRI), was found to possess mitochondrial superoxide dismutase (MnSOD)-mimetic activity and has been tested as a cytoprotectant [28]. The related compound calmangafodipir has been evaluated for neuroprotective effects in patients with colorectal cancer receiving oxaliplatin [29,30]. The development of calmangafodipir as a neuroprotectant for oxaliplatin was subsequently discontinued after the termination of two Phase III studies due to adverse hypersensitivity events. Nonetheless, preclinical studies have demonstrated that calmangafodipir can protect against oxaliplatin-induced small fiber neuropathy. Interestingly, this work has suggested a U-shaped effect of calmangafodipir, where a 5 mg/kg dose shows better effectiveness than either 2.5 mg/kg or 10 mg/kg, which is in agreement with previous observations [31]. In addition to calmangafodipir, carvedilol and rosmarinic acid have been evaluated for protective effects against oxaliplatin-induced neuropathic pain by preventing mitochondrial dysfunction and glial cell-mediated inflammation and may have a role in the treatment of CIPN [32,33].

Preclinical data also suggest promising activity associated with muscarinic toxin 7 (MT7), a muscarinic antagonist which can modulate Ca^2+^/calmodulin-dependent protein kinase β (CaMKKβ) to enhance the activity of AMP-activated protein kinase (AMPK), and peroxisome proliferator-activated receptor γ coactivator-1α (PGC-1α), to increase mitochondrial function and thus protect from neurodegeneration [34].

#### 2.1.4. Inflammatory Mediators

The activation of glial cells and the subsequent release and elevation of pro-inflammatory cytokines such as IL-1β, IL-6, TNF-α, and chemokines such as IL-8 and MCP-1 are common downstream events of neuropathic pain induced by chemotherapeutics [10,35]. One recent study reported that targeting IL-20 ameliorates paclitaxel-induced peripheral neuropathy by suppressing neuroinflammation and restoring Ca^2+^ homeostasis [36]. Furthermore, interference with the NCS-1/InsP_3_R/calpain cascade influences the production of IL-6 in DRG neurons, leading to the development of CIPN [37], suggesting a potential role of monoclonal antibodies in the prevention and treatment of CIPN. Chemokines and their receptors are also involved in the pathogenesis of CIPN. For example, induction of C-C chemokine ligand 2 (CCL2) and its receptor, C-C chemokine receptor 2 (CCR2) contributes to the development of oxaliplatin neurotoxicity, and blocking of this signal can attenuate the development of oxaliplatin-induced mechanical hyperalgesia [38].

The release of cytokines induced by chemotherapy might also result in activation of the Toll-like receptor (TLR) family member, especially TLR4 [39,40]. A recent study identified TLR9 signaling in macrophages as a new sexually-dimorphic biomarker that regulates chemotherapy-induced mechanical allodynia in male mice via neuron-macrophage interactions [41]. The implications of this observation for the development of more precise and effective therapeutics that could prevent and/or treat CIPN remain presently unclear.

### 2.2. Symptomatic Treatment-Based Therapeutics

Due to the incomplete knowledge about the underlying pathophysiology of CIPN, symptomatic treatments have not been consistently successful. It is known that neurotransmitters such as serotonin and norepinephrine are involved in the descending inhibitory nociceptive pathway and can amplify the effects of central sensitization [42]. Some studies have indicated that serotonin-norepinephrine reuptake inhibitors (SNRI), which inhibit the reuptake of these neurotransmitters and increase synaptic concentrations, can prevent input to the spinal dorsal horn neurons and thereby decrease pain transmission [42]. Currently, the only agent that has documented pharmacological activity in the treatment of CPIN is the SNRI, duloxetine [43]. Indeed, a meta-analysis on the effects of SNRI treatment of painful CIPN has confirmed both significant efficacy and safety for this indication [42]. Based on comparative trials, duloxetine was found to be more effective than venlafaxine in decreasing symptoms of CIPN, including motor neuropathy and neuropathic pain grade [44]. Previous studies suggested that the function of the descending inhibitory system by SNRIs relies on the concurrent action of both norepinephrine and serotonin [45]. Increasing only one neurotransmitter alone by either serotonin selective or norepinephrine selective reuptake inhibitors is less effective at producing analgesia than if both neurotransmitters are increased simultaneously [46,47,48]. As matter of fact, the more profound inhibition efficacy of duloxetine versus venlafaxine might partly attribute to its potent inhibition of both serotonin and norepinephrine reuptake, while venlafaxine has much less affinity for norepinephrine transporters [44,45]. Duloxetine is believed to act by inhibiting the activation of p38 phosphorylation, thus preventing the activation and nuclear translocation of the NF-kB transcription factor, reducing the inflammatory response, and inhibiting nerve injury by regulating nerve growth factor (NGF) without affecting the antitumor activity of oxaliplatin and paclitaxel [49]. However, the precise mechanism by which duloxetine affords neuroprotection is not entirely clear. For example, it has been reported recently that duloxetine acts as an inhibitor of OCT2 and thus may potentially restrict access of oxaliplatin to sites of injury within the peripheral nervous system [15]. Furthermore, duloxetine is known to inhibit poly ADP-ribose polymerase (PARP) cleavage, activate the tumor suppressor gene p53, and affect the Bcl2 family to reverse paclitaxel-induced oxidative stress and apoptosis [50].

In addition to SNRIs, several other neurological agents have been evaluated to treatment drug-induced neuropathic pain, including gabapentin and pregabalin, antiepileptic drugs that are structurally related to the neurotransmitter gamma-aminobutyric acid (GABA). The mechanism of action of these agents is based on selective binding to the α2δ subunit of the voltage-dependent calcium channel (VDCC), which is thought to play an important role in neuropathic pain [51]. Both gabapentin and pregabalin, given alone or in combination with opioids, have been used to prevent or treat CIPN in clinical trials, however, the results did not support that they help prevent or decrease the CIPN [52,53]. Although a small study (20 patients per arm) evaluating gabapentin 300 mg 3 times a day in a double-blind, randomized trial in patients receiving paclitaxel reported a significant reduction in CIPN, confirmation of this is needed in a subsequent trial [54]. This is consistent with the ASCO CIPN guideline, which states that outside the context of a clinical trial, gabapentin or pregabalin is not indicated as a preferred intervention for the treatment of CIPN.

### 2.3. Other Strategies

Besides pathologic mechanism and symptomatic treatment-based therapeutics, several additional strategies have been proposed for the prevention and/or treatment against CIPN for which preliminarily supporting evidence has been generated.

#### 2.3.1. Herbal Medicines

Several herbal medicines have generated interest, particularly among patients, as a potentially safe way for alternative intervention, and for some of these, a potential to prevent or treat CIPN has been documented. However, uncertainties regarding the use of single herbal medicines compared with the combination of multiple herbs, the absence of unequivocal information on the identity and content of active ingredients, and the lack of information on the mechanism of action have hindered proper clinical evaluation of their effectiveness [55]. Nonetheless, both single herbal medicines, as well as mixed herbal formulas, have been evaluated for the prevention and/or treatment for CIPN associated with platinum-based drugs [56,57], taxanes [58,59], and vinca alkaloids [60,61] in animal models, “Goshajinkigan” (GJG), a Kampo medicine, has been the most frequently studied herbal product for the prevention of CIPN in clinical trials [62,63,64]. However, a multicenter randomized Phase III trial comparing the combination fluorouracil, leucovorin, and oxaliplatin (mFOLFOX6) with and without GJG failed to meet the primary endpoint [63]. GJG is a mixture of aqueous extracts of 10 crude herbs in fixed proportions, each of which containing numerous ingredients. Two mechanisms have been suggested by which GJG may alleviate peripheral neurotoxicity, one is the promotion of the release of dynorphin, and the other one is the promotion of nitric oxide production. While GJG may prevent acute mild neuropathy and allow an increase in the dose intensity of oxaliplatin, the net result may be an increase in severe neuropathy, and this ultimately resulted in the discontinuation of the Phase III study [63,65]. Despite these discouraging results, additional more herbal medicines are currently being evaluated in exploratory clinical trials to suppress CIPN [66,67].

#### 2.3.2. Monoclonal Antibody and Gene Therapeutics

Although research conducted over the last few years has offered new mechanistic insights into the etiology of CIPN and provided a foundation for novel promising strategies targeting a variety of targets/pathways, the lack of small-molecule lead candidate drugs for several of the identified targets has hampered rapid translation to the clinic. For example, several reports have suggested that inhibiting SARM1 is a promising strategy to reduce pathological axonal degeneration induced by chemotherapeutic drugs [22,68], no approved drugs exist that are known to target SARM1. This has resulted in alternative approaches in which investigators developed in vivo gene therapeutics to block pathological axon degeneration by inhibiting SARM1, an approach that could possibly be used clinically to treat manifold neurodegenerative diseases characterized by axon loss [23].

Similarly, derived from clinical studies demonstrating that increased levels of interleukin-6 (IL-6) and soluble IL-6 receptor correlates with painful CIPN [69], preclinical studies have been performed using an IL-6-neutralizing antibody to prevent the prevention of paclitaxel-induced neuropathy in mice, providing a direct rationale for a clinical trial with siltuximab (IL-6 neutralizing antibodies) to prevent CIPN [37]. Another study indicated that a monoclonal antibody targeting the matrix metalloproteinase 9 (MMP9) can prevent and reverses paclitaxel-induced peripheral neuropathy in mice, suggesting that andecaliximab (GS-5745, an MMP9 monoclonal antibody) may offer new therapeutic approaches for the treatment of CIPN [70].

#### 2.3.3. Gut Microbiota Mediated Strategy

It is well known that gut microbiota plays a critical role in the absorption, elimination, efficacy, and toxicity of many xenobiotics [71,72,73]. In addition, changes in the gut microbiota can modulate both the peripheral and central nervous systems and result in altered brain functioning through the “microbiota gut–brain axis” [74]. Preclinical studies have demonstrated that gut microbiota promotes the development of chemotherapy-induced mechanical hyperalgesia [75,76]. In particular, it has been reported that oxaliplatin-induced mechanical hyperalgesia can be diminished in germ-free mice and mice pretreated with antibiotics and after the restoration of the microbiota in germ-free mice, this protective effect could be reversed [76]. Interestingly, a mouse-strain-dependent peripheral neuropathy phenotype has been documented for paclitaxel, and this was found to be largely dependent on strain differences in gut microbiota diversity [75]. While several investigations have reported relationships between gut microbiota and CIPN, the mechanism by which the gut microbiota affects sensitivity to CIPN remains incompletely understood. One possible mechanism involves TLR4 signaling, where stimulation of TLR4 by LPS derived from gram-negative bacteria can result in activation of NF-κB and subsequent production of pro-inflammatory cytokines, chemokines, and enzymes [77].

In the context of cancer chemotherapy, it should be pointed out that the gut microbiota homeostasis could be changed by the drugs themselves at both the phylum and genus levels. For example, levels of *Lactobacillus* and *Bifidobacterium* are reduced in subjects receiving chemotherapy compared with controls [77], and this suggests that probiotics intervention can target dysregulated microbiota during treatment with chemotherapeutic drugs for the prevention or treatment of CIPN. Therefore, targeting the gut microbiota might be a possible strategy that could provide a more personalized cancer treatment [78]. It is worth noting that, since gut microbiota can indirectly impact the metabolism of drugs, intentional modulation of the gut microbiome might result in altered pharmacokinetic profiles of chemotherapeutic drugs, and lead to unwanted changes in drug efficacy and/or other toxicities. At this time, there are insufficient data to recommend such an approach for the prevention or treatment of CIPN.

## 3. Non-Pharmacological Interventions for Prevention or Treatment of CIPN

There is currently increasing interest in non-pharmacological strategies for the prevention or treatment of CIPN due to the anticipation that avoidance of drugs may allow for benign intervention approaches. However, phase III evidence of benefit for several of these approaches, including acupuncture, physical exercise, cryotherapy/compression, and scrambler therapy is not yet available, and further research in larger clinical trials is needed to better delineate their utility.

### 3.1. Acupuncture

Acupuncture therapy, associated with Traditional Chinese Medicine, has a long history of treating pain. Acupuncture is well-accepted and safe, and adverse effects are quite rare [79,80]. One small randomized, sham-controlled trial of weekly electro-acupuncture for the prevention of taxane-induced peripheral neuropathy in 63 patients did not show any differences in neuropathy between groups [81]. In contrast, as an alternative and complementary therapy, acupuncture has demonstrated benefits in treating peripheral neuropathies and cancer-related pain [82,83]. Data from emerging trials also demonstrate that acupuncture may be beneficial for certain types of CIPN [84]. A recent randomized controlled pilot trial to assess the feasibility, safety, and effects of an acupuncture intervention on CIPN of breast cancer survivors was conducted [85]. In this trial, 8-week acupuncture intervention, versus usual care, led to clinically meaningful and statistically significant improvements in neuropathic sensory symptoms in breast cancer survivors with mild and moderate CIPN after the completion of chemotherapy, no serious side effects were observed [85].

Additional larger studies with higher quality clinical data are needed to confirm the effect of acupuncture therapy on CIPN. Based on the existing experience, acupuncture therapy protocols in different trials vary substantially and are practitioner-dependent, and the use of placebo and/or sham treatments to control for bias are inconsistent. Establishing “gold standard” acupuncture protocols, placebos, and outcomes for peripheral neuropathy is critical to get a more reliable, objective assessment of acupuncture therapy [80].

### 3.2. Cryotherapy and Compression Therapy

In recent years, the efficacy of cryotherapy and compression therapy to prevent taxane-induced peripheral neuropathy has been reported [86,87]. Cryotherapy was considered as a potentially efficient strategy for the prevention of CIPN because it is well-tolerable and because no serious adverse effects are observed [88]. Several clinical trials also revealed that compression therapy using surgical gloves is a safe and potentially effective therapy for the amelioration of CIPN [87,89]. However, a recent study prospectively compared the efficacy of cryotherapy and compression therapy for CIPN and found no difference in the incidence of nab-paclitaxel (Abraxane) -induced peripheral neuropathy using either cryotherapy or compression therapy [90]. Additional large-scale research is on-going to sort out the utility and toxicities associated with cryotherapy, compression therapy, and/or cryo-compression therapy, for the prevention of CIPN [91].

### 3.3. Scrambler Therapy

Scrambler therapy is a cutaneous neuro-stimulatory treatment that has been utilized for the treatment of several chronic pain syndromes [92,93] and the management of CIPN [94]. A recent randomized Phase II pilot trial was conducted to evaluate the effect of scrambler therapy for treating CIPN [95]. Patients were randomized to receive scrambler therapy versus transcutaneous electrical nerve stimulation (TENS) for 2 weeks. Compared with TENS treated patients, scrambler therapy treated patients had at least a 50% documented improvement during the 2 treatment weeks, suggesting a promising potential of scrambler therapy for treating CIPN. To further confirm the effect of scrambler therapy on the treatment of CIPN, larger, placebo-controlled, double-blinded clinical trials to estimate the effectiveness of scrambler therapy should be considered. In addition, further exploration of the mechanism by which scrambler therapy exerts its efficacy is needed.

### 3.4. Physical Exercise

Several studies have suggested that exercise may be beneficial for other types of peripheral neuropathy and in treating other cancer treatment-associated symptoms such as pain, fatigue, mood, emotional distress, sleep disturbance, balance, and decreased quality of life [96,97,98]. Physical exercise may attenuate CIPN through its influence on blood circulation/oxidative stress, inflammation, neurotransmitters, endogenous opioids, growth factors, neuroplasticity, and coping and symptom interaction mechanisms [99]. A single-blind, randomized controlled exploratory study compared standard of care to a physical-therapy home program (4 visits) throughout adjuvant taxane chemotherapy for stage I-III patients with breast cancer was conducted [100]. In this study, the treatment group showed strong trends toward less pain, and pain decreased over time, pain pressure thresholds and grip dynamometry were also improved. This supports the possibility that a physical-therapy home program may improve CIPN pain in the upper extremity for patients with breast cancer who are receiving neurotoxic chemotherapy.

### 3.5. Photobiomodulation

Photobiomodulation (PBM) is a new and emerging therapeutic tool involving nonionizing, low-power, laser light therapy in the supportive care of cancer patients [101]. A randomized, sham-controlled clinical trial was conducted for evaluating the effect of PBM on CIPN [102]. PBM patients experienced a significant reduction in modified total neuropathy scores at 4, 8, and 16 weeks after initiating treatment, and PBM produced a significant reduction in neuropathy symptoms. Despite these positive findings, at this stage, consideration of PBM should be made with extreme caution as evidence is limited and adverse effect profiles have not yet been adequately determined [103].

## 4. Conclusions

CIPN is a common and persistent complication of commonly used chemotherapy drugs. The empiric identification of agents and interventions to mitigate CIPN has been disappointing, and presently there is no intervention available for prevention, and only one agent (duloxetine) has shown moderate evidence of treatment efficacy. Considering the debilitating consequences of CIPN on quality of life, it is imperative that future studies focus on details of events and biological pathways leading up to CIPN and that the development of effective clinical interventions should be derived from these mechanistic insights. Furthermore, the development, use, and further refinement of appropriately predictive non-clinical models are urgently needed as this will provide a critical first step in ultimately identifying safe and effective treatments for prevention or intervention. Indeed, it is likely that the failure to provide reproducible approaches and the current absence of effective strategies to combat CIPN is at least partially related to the prior use of model organisms without consideration of strain differences, age- and sex-dependence of phenotypes of interest, as well as the use of unstandardized behavioral tests, a failure to apply adequately-powered study designs that include appropriate controls and randomization.

Despite these study design problems, substantial progress has been made in recent years in our understanding of the pathogenesis for CIPN, and this work has provided important new mechanistic insights and a rationale for novel pharmacological and non-pharmacological strategies (Figure 1). Several of these emerging therapeutic strategies have focused on targeting neuronal transporters, neuroprotective mechanisms, neuroinflammation, mitochondrial enzymes and oxidative stress, serotonin-norepinephrine reuptake, and nociceptor sodium channel inhibition, and many of these approaches are now under clinical evaluation (Table 1). These clinical studies provide additional challenges that require careful consideration and include a selection of eligibility criteria, selection of outcome measures and endpoints, potential effects of the intervention on the efficacy of chemotherapy, and statistical issues related to sample sizes of randomized groups based on anticipated effect size and variability in the primary endpoints. In light of these considerations, we contend that the future development of improved, efficient intervention strategies for CIPN requires a developmental, collaborative, reverse-translational strategy that involves a multidisciplinary team of experienced pharmacologists, statisticians, and oncologists. Ultimately, such advances will help guide the care of millions of cancer survivors who are suffering from CIPN and its quality of life sequela.

## Author Contributions

Conceptualization, all authors; manuscript writing, Y.L., M.B.L., and S.H.; supervision, M.B.L. and S.H. All authors have read and agreed to the published version of the manuscript.

## Figures and Tables

**Table 1:** List of emerging clinical trials for the prevention and treatment of chemotherapy-induced peripheral neuropathy (CIPN).

NCT #	Intervention	Phase	N	Patient Population	Target/Pathway
NCT04205903	Nilotinib	Phase 1b Phase 2	90	Patients with stage I–III breast cancer receiving paclitaxel therapy	Prevention, OATP1B1-3 uptake transporter inhibitor
NCT04164069	Dasatinib	Phase 1	20	Patients with stage IV colorectal cancer receiving FOLFOX chemotherapy and bevacizumab	Prevention, OCT2 uptake transporter inhibitor
NCT04034355	Calmangafodipir	Phase 3	301	Patients with colorectal cancer indicated for adjuvant modified FOLFOX6 (mFOLFOX6) chemotherapy	Prevention, reduction of ROS
NCT03654729	Calmangafodipir	Phase 3	289	Patients with metastatic colorectal cancer (mCRC) indicated for first-line modified FOLFOX6 (mFOLFOX6) chemotherapy	Prevention, reduction of ROS
NCT04201561	Sodium selenite pentahydrate	Phase 3	68	Platinum-sensitive recurrent ovarian, fallopian, or primary peritoneal cancer patients	Prevention, reduction of ROS
NCT04112641	Nicotinamide riboside	Phase 2	142	Cancer survivors who have completed chemotherapy with taxane or platinum-complex compounds	Prevention, increase levels of NAD^+^
NCT03642990	Nicotinamide riboside	Phase 2	39	Patients receiving paclitaxel or nab-paclitaxel for the treatment of metastatic breast cancer or ovarian, endometrial, peritoneal, or fallopian tube cancer	Prevention, increase levels of NAD^+^
NCT04492436	Thrombomodulin alfa (ART-123)	Phase 2	300	Patients with unresectable metastatic colorectal cancer who receive oxaliplatin-containing chemotherapy	Prevention, anti-coagulation, anti-inflammatory, and anti-fibrinolytic effects through activated TAFI
NCT03722680	Riluzole	Phase 2	210	Stage II/III colorectal cancer patients received simplified FOLFOX4	Prevention, activating TREK/TRAAK channels
NCT03688633	Candesartan	Phase 2	40	Patients with non-Hodgkin’s type B malignant lymphoma with multidrug therapy containing vincristine	Prevention, angiotensin II type 1 receptor antagonist
NCT03254394	Lidocaine hydrochloride	Phase 1 Phase 2	38	Patients with colorectal cancer treated with the FOLFOX chemotherapy	Prevention, sodium channel blocker
NCT04137107	Duloxetine	Phase 2 Phase 3	327	Patients with stage II-III colorectal cancer suffering peripheral neuropathy caused by treatment with oxaliplatin	Prevention, serotonin–norepinephrine reuptake inhibitor
NCT03812523	Duloxetine, Lorcaserin	Phase 2	50	Cancer patients receiving oxaliplatin treatment reporting neurotoxicity Grade 2	Treatment, serotonin–norepinephrine reuptake inhibitor
NCT02271893	Dextromethorphan	Phase 2	40	Breast cancer patients suffering from CIPN for at least 3 months after the end of their last cancer chemotherapy	Treatment, NMDA receptor antagonists
NCT03709888	Memantine XR-pregabalin		20	Patients with chemotherapy-induced peripheral neuropathy	Treatment, NMDA receptor antagonist and gabapentinoids
NCT03571334	Botulinum Toxin A	Phase 2	40	Been diagnosed with breast cancer and undergone treatment with taxane-based chemotherapeutic agents.	Treatment, inhibiting the release of neurotransmitters
NCT04282590	TRK-750	Phase 2	240	Oxaliplatin-containing chemotherapy treatment for colorectal cancer	Treatment
NCT03943498	Fingolimod	Phase 1	10	Pain or symptoms of CIPN	Treatment, S1PR1 antagonism
NCT04205071	Lorcaserin	Phase 1	30	Patients with gastrointestinal (GI) cancer or breast cancer stages I-IV prior exposure to paclitaxel or oxaliplatin	Treatment, selective serotonin (5-HT) agonist
NCT03782402	Cannabinoids	Phase 2	100	Breast cancer patients experiencing TIPN due to paclitaxel or docetaxel	Treatment, targeting the endocannabinoid system
NCT04398446	Hemp-based cannabidiol	Phase 2	100	Non-metastatic breast, colorectal, uterine, and ovarian cancer patients included neurotoxic chemotherapeutic agents	Treatment, 5-HT_1A_ receptor system
NCT04299893	Ozone	Phase 2 Phase 3	42	Cancer of colon and rectum in any stage, with treatment including oxaliplatin	Treatment, controlled oxidative stress
NCT04468230	Nicotine transdermal patch	Phase 2	40	Clinically diagnosed peripheral sensory neuropathy	Supportive care, nicotinic acetylcholine receptors, ion channels
